# Complete remission in metastatic primary malignant melanoma of the esophagus with nivolumab: a case report

**DOI:** 10.1186/s13256-021-02928-w

**Published:** 2021-07-14

**Authors:** Takeshi Okamoto, Eriko Nakano, Teruo Yamauchi

**Affiliations:** 1 Department of Gastroenterology, St. Luke’s International Hospital, 9-1 Akashicho, Chuo-ku, Tokyo, 104-8560 Japan; 2 Department of Oncology, St. Luke’s International Hospital, 9-1 Akashicho, Chuo-ku, Tokyo, 104-8560 Japan

**Keywords:** Primary malignant melanoma of the esophagus, Immune checkpoint inhibitor, Nivolumab, Bladder tumor, Case report

## Abstract

**Background:**

Primary malignant melanoma of the esophagus is a rare form of mucosal melanoma with a poor prognosis. While immune checkpoint inhibitors have recently extended overall survival in metastatic melanoma, data on their effects on primary malignant melanoma of the esophagus are limited because of its rarity. Here, we report the first case of long-term complete remission of metastatic primary malignant melanoma of the esophagus after nivolumab monotherapy.

**Case presentation:**

A 79-year-old Asian man with a history of prostate cancer, gallbladder cancer, deep vein thrombosis, hypertension, and diabetes mellitus presented with gross hematuria. Cystoscopy revealed a solitary tumor on the right posterior wall of the bladder, and transurethral resection of bladder tumor was performed. Pathology was consistent with metastatic melanoma. A pigmented submucosal tumor-like growth in the esophagus was discovered on esophagogastroduodenoscopy. Computed tomography showed widespread metastases. The patient was diagnosed as having primary malignant melanoma of the esophagus with metastases to the stomach, subcutaneous tissue, lung, bladder, pleura, and peritoneum. Complete remission was achieved after seven cycles of triweekly nivolumab monotherapy. While nivolumab was discontinued because of kidney injury, the patient has remained tumor-free for over 4 years without further treatment.

**Conclusion:**

Immune checkpoint inhibitors may have astonishing curative effects in selected populations. More research is warranted to identify factors that increase the likelihood of achieving complete remission in primary malignant melanoma of the esophagus as well as in other melanomas.

## Background

More than 95% of melanomas arise in the skin, with the eye being the second most common site. Mucosal melanomas account for less than 1% of all malignant melanomas [1]. Primary malignant melanoma of the esophagus (PMME) accounts for about 2% of head and neck mucosal melanomas [2]. PMME is estimated to account for 0.1–0.2% of esophageal malignancies, generally occurs in the sixth and seventh decades of life, and is twice as common in men than in women [3–5]. About half of PMME patients have metastases at the time of diagnosis [5].

While PMME has a poor prognosis with median overall survival of 10–14 months, the advent of immune checkpoint inhibitors (ICI) has improved survival in malignant melanoma patients and may offer similar benefits to PMME patients [4, 6]. Here, we present the first case of metastatic PMME in which complete remission was achieved after nivolumab monotherapy and sustained for over 4 years after discontinuing nivolumab.

## Case presentation

A 79-year-old Asian man presented with gross hematuria that started 2 weeks prior. His morning urine was bright red when he began to urinate, after which it grew progressively lighter in color. His urine later in the day was pink and sometimes yellow or colorless at night. He noted mild weight loss despite normal appetite and food intake. He also complained of mild cough and slight dyspnea upon exertion.

He had a history of open cholecystectomy for gallbladder cancer, radical prostatectomy with penile prosthesis implantation for prostate cancer, inferior vena cava filter placement for deep vein thrombosis, hypertension, and diabetes mellitus. Medications included edoxaban, an angiotensin II receptor blocker, a dipeptidyl peptidase-4 inhibitor, and a proton pump inhibitor. He was an occasional drinker and had quit smoking 40 years ago. No significant family history was noted. He had opted to receive annual medical checkups including serum and urine testing with tumor markers, chest roentgenogram, abdominal ultrasound, and esophagogastroduodenoscopy (EGD) for over 30 consecutive years. No abnormalities were noted at the last checkup, which was conducted 11 months prior to his presentation.

**Fig. 1:**
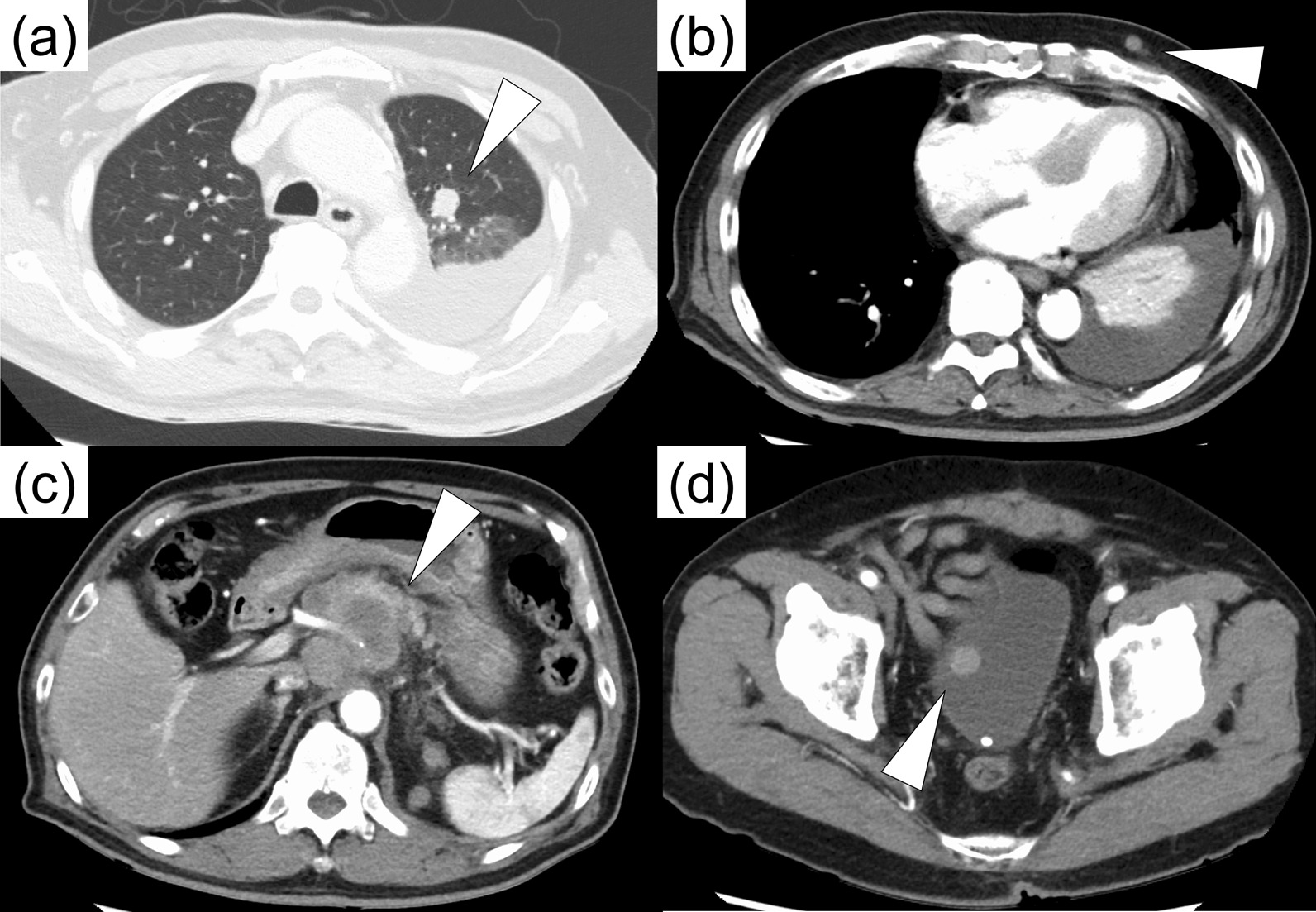
Contrast computed tomography revealed tumors (white arrowheads) in the left lung (**a**), subcutaneous tissue (**b**), abdominal lymph nodes (**c**), and bladder (**d**) consistent with metastatic disease

Physical examination was significant only for abdominal surgical scars and decreased breath sounds on the left side. Laboratory testing revealed an increase in white blood cells (10,200 mm^3^) and C-reactive protein (6.76 mg/dL). Neuron-specific enolase was elevated (30.7 ng/mL), while other tumor markers such as carcinoembryonic antigen, squamous cell carcinoma antigen, and prostate-specific antigen were within their normal ranges. Urinalysis showed over 50 red blood cells per high-powered field, and urine cytology showed severe atypia, raising the suspicion of urothelial carcinoma. Contrast computed tomography (CT) showed a well-enhanced bladder tumor with no signs of deep invasion. Severely enlarged abdominal lymph nodes and lung, pleural, and peritoneal metastases were also observed. A small subcutaneous nodule in the left thorax was also suspected to be a metastatic lesion. While metastatic urothelial carcinoma was a possible explanation, a coexisting cancer of unknown origin was also suspected (Fig. 1).

**Fig. 2:**
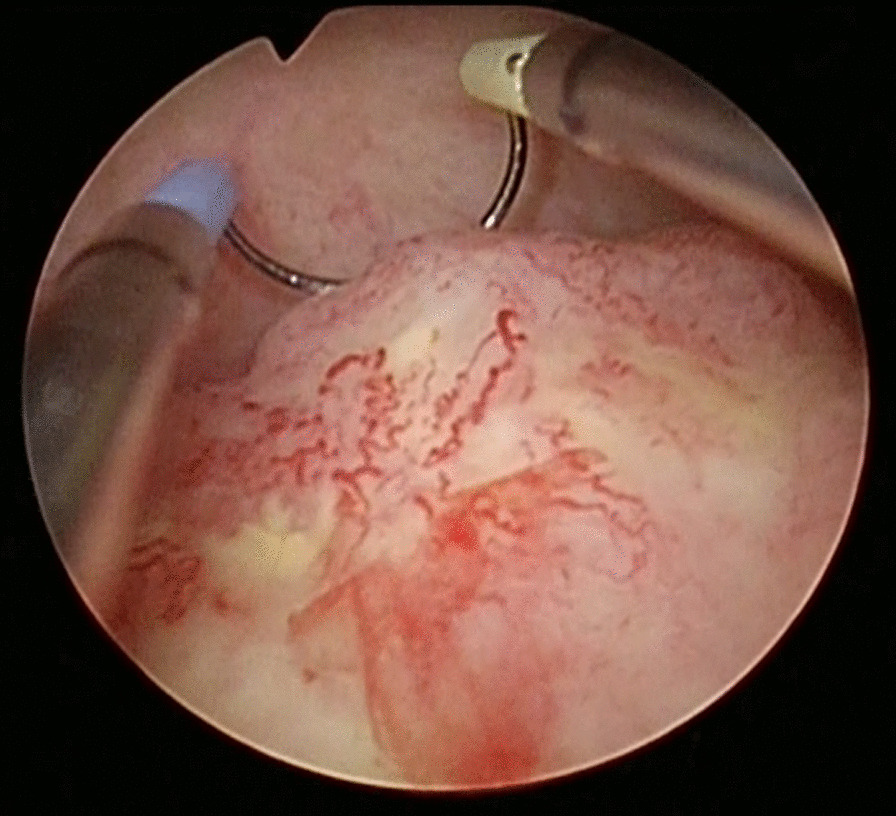
A 15-mm white mass was observed in the right posterior wall of the bladder and removed by transurethral resection of bladder tumor

**Fig. 3:**
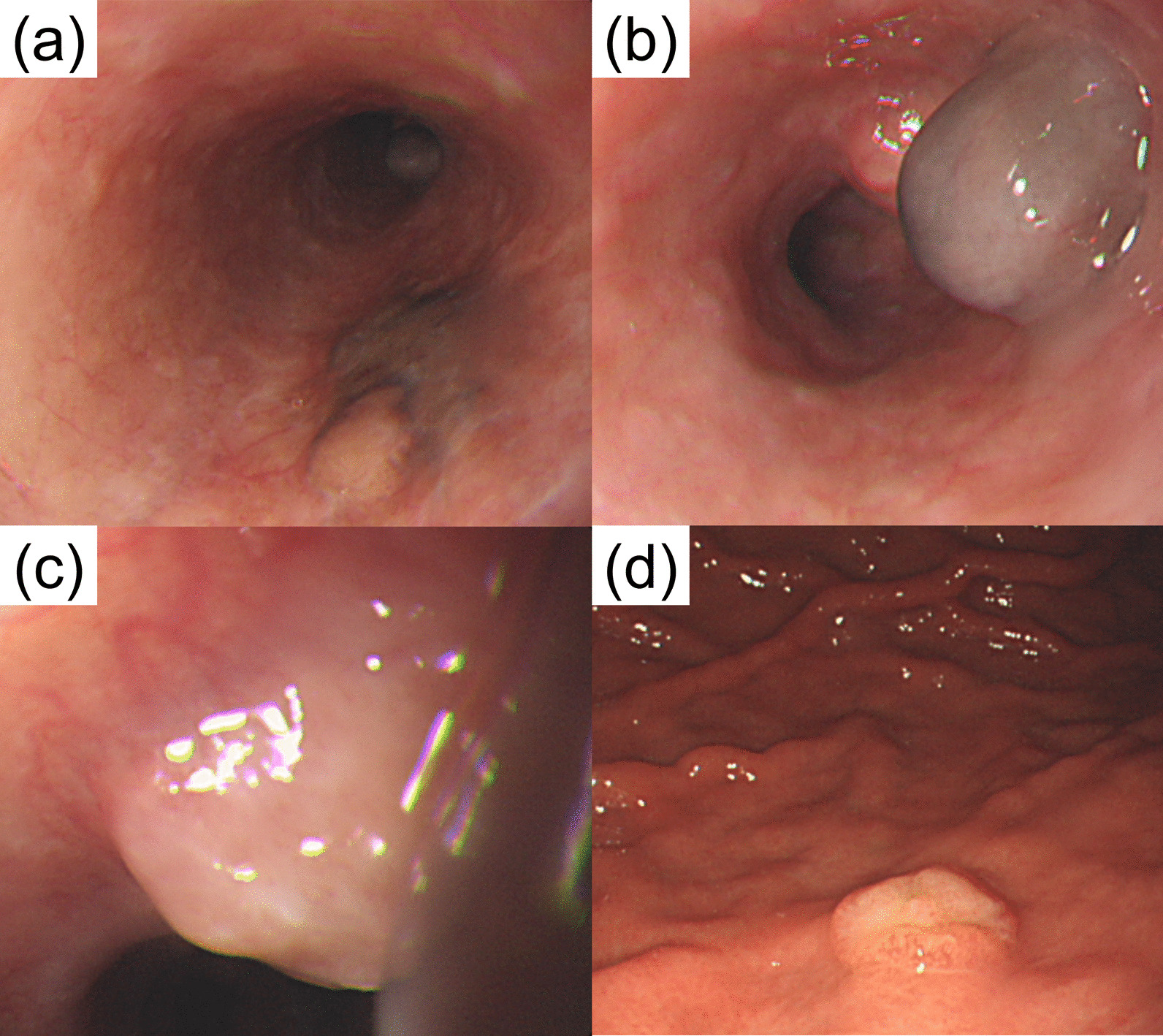
Esophagogastroduodenoscopy revealed a 20-mm flat, pigmented lesion with a nonpigmented nodule in the mid-esophagus (**a**), a 10-mm pigmented submucosal tumor-like growth in the distal esophagus (**b**), and polypoid lesions with central depression in the distal esophagus (**c**) and stomach (**d**). All were confirmed to be malignant melanoma lesions on pathology

Cystoscopy revealed a 15-mm white mass on the right posterior wall of the bladder (Fig. 2). Transurethral resection of bladder tumor (TUR-BT) was performed with no complications. While macroscopic findings of the bladder tumor appeared consistent with those of urothelial tumors, further testing was conducted to rule out concomitant malignancies. EGD revealed a 20-mm flat, pigmented lesion with a nonpigmented nodule in the mid-esophagus, a 10-mm pigmented submucosal tumor-like growth in the distal esophagus, and polypoid lesions with central depression in the distal esophagus and stomach (Fig. 3). Endoscopic ultrasound-guided fine needle aspiration of an abdominal lymph node and thoracentesis were also performed. Colonoscopy was unremarkable.

**Fig. 4:**
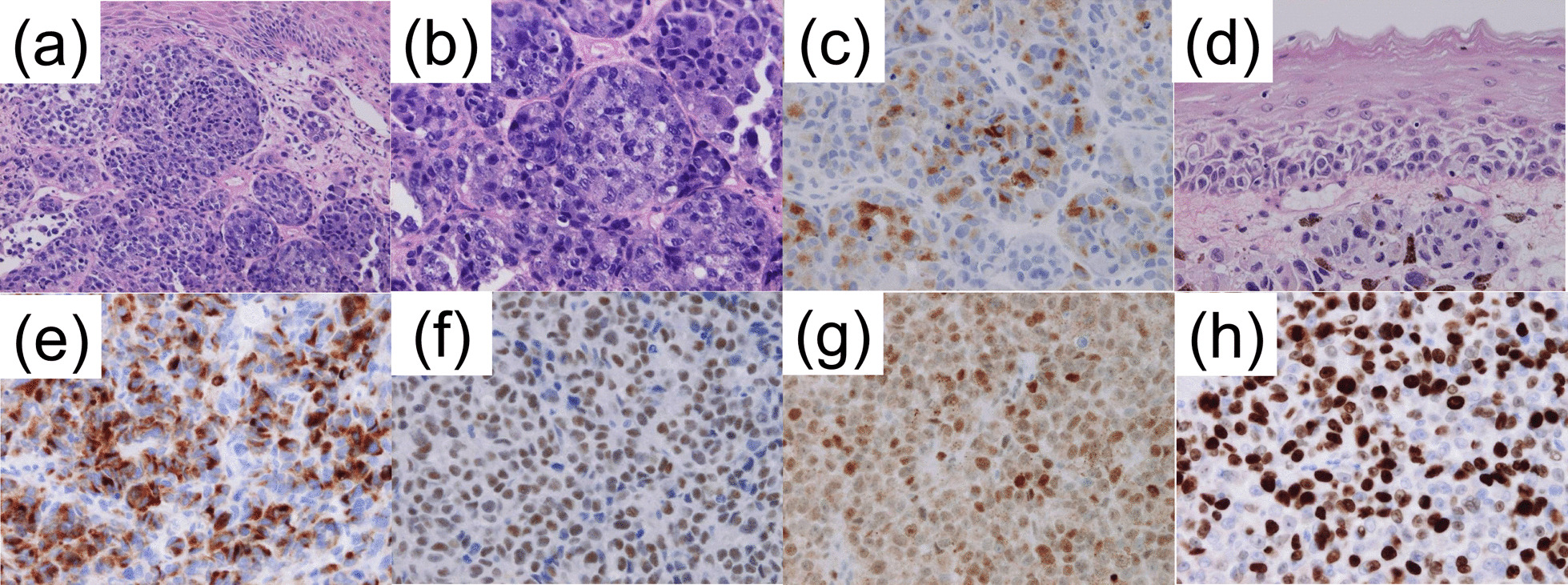
Low-power (**a**) and high-power (**b**) magnification of esophageal biopsies showed melanin granules within tumor cells and melanoblasts in the interstitium. Immunohistochemistry was positive for melan A (**c**). *In situ* lesions were also observed, suggesting that the esophagus was the primary site (**d**). Immunohistochemistry for the resected bladder specimen was positive for melan A (**e**), SOX10 (**f**), and p53 (**g**). The MIB-1 proliferation index was 80% (**h**)

Esophageal biopsy showed melanin granules within tumor cells and melanoblasts in the interstitium, with immunohistochemistry positive for melan A (Fig. 4a–c). *In situ* lesions were also observed, suggesting that the esophagus was the primary site (Fig. 4d). Stomach biopsy revealed similar proliferation of tumor cells. Pathology of the bladder tumor revealed no signs of muscular invasion. Immunohistochemistry for both the bladder tumor and lymph node was positive for melan A, vimentin, S-100, HMB45, SOX10, and p53 and negative for AE1/AE3 (Fig. 4e–h). The MIB-1 proliferative index was 80%. A negative test for the EWSR1 chimeric gene ruled out clear cell sarcoma. No BRAF mutation was found. Comprehensive dermal and ocular examinations were negative for melanoma. The patient was diagnosed with PMME with metastases to the stomach, subcutaneous tissue, lung, bladder, pleura, and peritoneum.

**Fig. 5:**
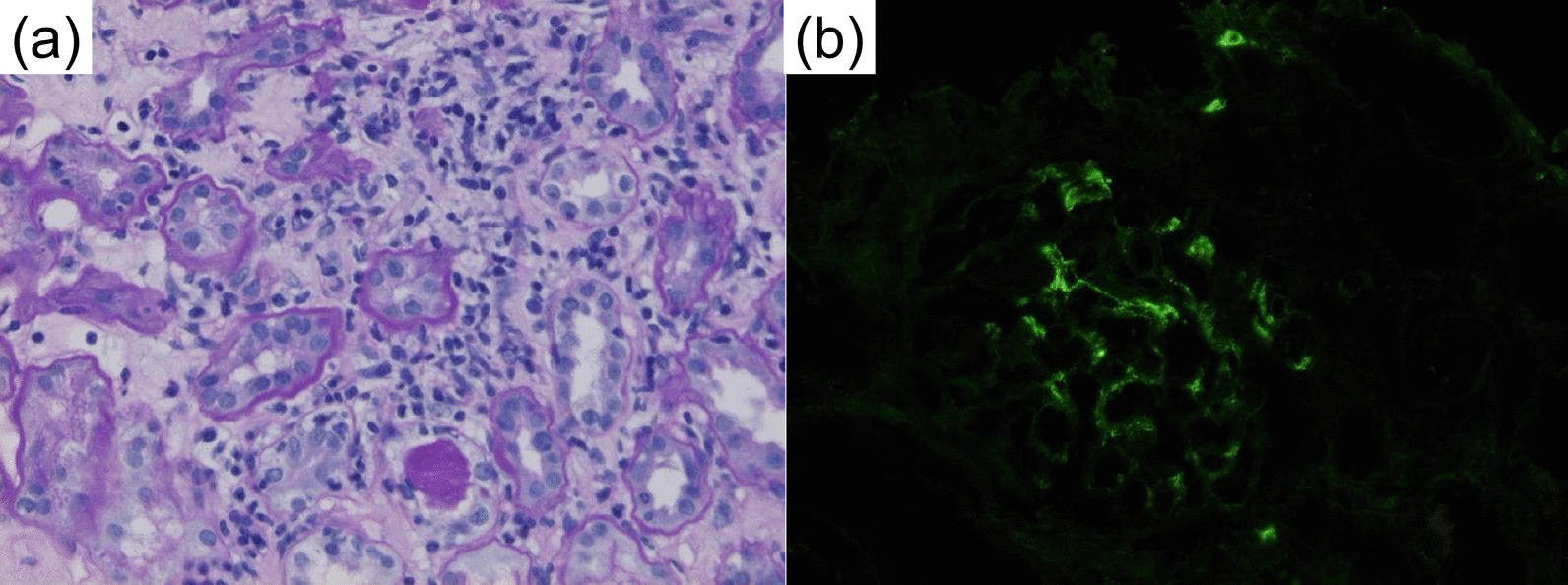
Periodic acid–Schiff staining (**a**) and IgA immunofluorescence microscopy (**b**) of the right kidney, suggestive of mild tubulointerstitial nephritis and IgA nephropathy

**Fig. 6:**
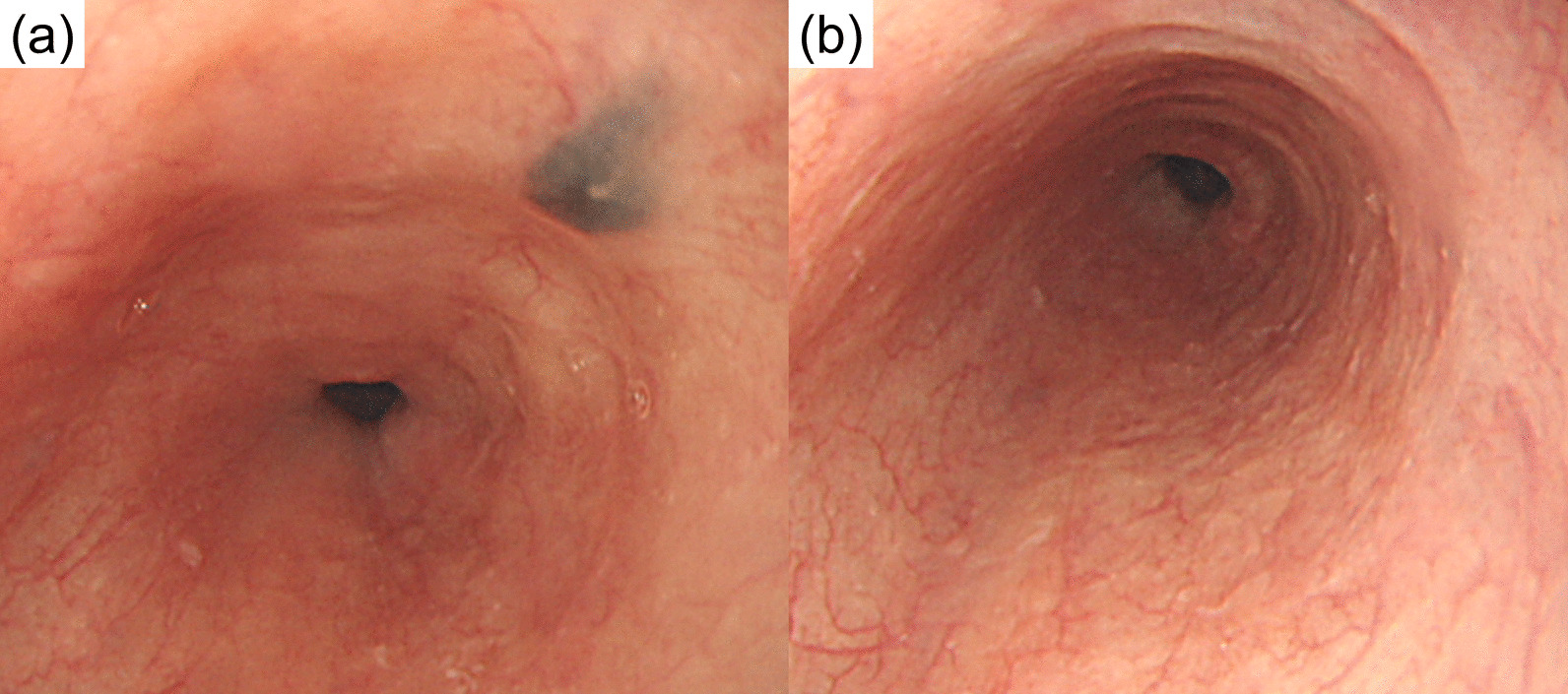
All esophageal and gastric tumors had disappeared 1 year after nivolumab treatment, leaving only slight melanosis with no remaining pathological evidence of malignancy (**a**). The melanosis also disappeared 6 months thereafter (**b**)

Nivolumab was started at 2 mg/kg every 3 weeks, which was the standard dosage at the time. CT follow-up after six cycles revealed complete remission of all visible tumors. However, acute kidney injury was observed after seven cycles. Pathology from renal biopsy was suggestive of both mild interstitial tubulonephritis and IgA nephropathy (Fig. 5). Partial recovery was achieved after discontinuing nivolumab and introducing steroids at 0.5 mg/kg, which were tapered over a 3 month period. While nivolumab therapy was not reintroduced, repeated CT scans taken every 3–6 months also showed no signs of recurrence. Follow-up endoscopy 1 year later revealed that all esophageal and gastric tumors had disappeared, leaving only slight melanosis with no remaining pathological evidence of malignancy (Fig. 6a). The melanosis also disappeared 6 months thereafter (Fig. 6b). The patient has remained tumor-free for over 5 years without further treatment.

## Discussion and conclusions

Since the first report by Baur in 1906, over 300 PMME cases have been reported to date [6]. Risk factors for PMME have not been elucidated; those associated with cutaneous melanoma such as sun exposure have no connection with PMME. Over 90% are found in the middle and distal thirds of the esophagus. PMME lesions are usually polypoid and pigmented but can take a variety of forms and are amelanotic in 10–25% of cases [7]. Diagnosis is therefore based on histology, as defined by Allen and Spitz in 1953: a) typical melanoma histology and melanin granules within tumor cells, b) origination from epithelium with junctional activity, and c) junctional activity with melanotic cells in adjacent epithelium [8, 9]. Diagnosis can present challenges even with the use of immunohistochemistry markers such as S-100 (the most sensitive), melan-A (the most specific), and HMB-45 [10]. Metastatic melanoma to the esophagus is rarer than PMME.

Surgery has traditionally been the only option to prolong survival in melanoma, as efficacy of both chemotherapy and radiotherapy are limited. The introduction of programmed death 1 (PD-1) inhibitors nivolumab and pembrolizumab as well as the cytotoxic T-lymphocyte-associated antigen 4 inhibitor ipilimumab in combination with nivolumab significantly extended overall survival in metastatic melanoma [11–13]. ICIs are positioned as first-line therapy for melanoma without BRAF mutations and 3 year overall survival rates have reached 51%, 40%, and 56%, respectively [14].

While nivolumab has been shown to be less effective in mucosal melanomas than in cutaneous melanomas, the standard treatment and the efficacy of ICI in PMME remains unclear due to its rarity [15]. The largest study evaluating ICI treatment in PMME included a cohort of 12 patients treated with PD-1 inhibitors in a retrospective analysis of 76 unresectable or metastatic PMMEs [16]. The study revealed promising results, with 75% achieving partial response and the other 25% maintaining stable disease for at least 4 months. The mean progression-free survival was 15.6 months and severe toxicity was only seen in 1 patient. While case reports of ICI treatment for PMME are scarce, one case of unresectable PMME maintained good partial response after 30 courses of nivolumab monotherapy, while another had stable disease for 11 cycles [17, 18]. Another PMME case refractory to chemoradiotherapy showed good partial response after 3 cycles of nivolumab but discontinued treatment after 7 cycles due to irAEs, ultimately resulting in tumor death [19]. Our case is the first report of complete remission in metastatic PMME with nivolumab monotherapy.

Despite a large representation of the elderly in the cancer population, they are almost always underrepresented in clinical trials. ICI treatment was expected to have less efficacy on elderly patients due to immunosenescence, or reduced immune function due to continuous remodeling of lymphoid organs which increase the incidence of infections, neoplasia, and autoimmune disease [20]. Similarly, the elderly were expected to experience more immune-related adverse events (irAEs). However, meta-analyses of ICI treatment showed no significant difference in efficacy between the younger and older groups with cut-offs of 65, 70, and 75 years of age [21, 22]. Frequency and severity of irAEs were also similar between the younger and elderly groups [23]. Efficacy and irAE profile independent of age were also seen in various ICI studies limited to melanoma patients [24–26]. In fact, a large study with 538 metastatic melanoma subjects showed that patients above the age of 60 experienced higher efficacy with anti-PD1 therapy than their younger counterparts, with the chance of disease progression decreasing by 13% for every decade of life [27]. The authors reproduced the result in mice and proposed that a change in the regulatory T cell composition with age may help explain this higher efficacy. While immunosenescence may lead to decreased immune function, the elderly also have increased chronic inflammation which suggests an augmented immune function. Age may have played a role in the complete remission of our 79 year-old patient.

It is also possible that spontaneous regression, where histologically-proven cancer regresses without sufficient therapy to explain it, also played a role. Melanomas, renal cell carcinomas, and neuroblastomas are among the most reported malignancies which regress spontaneously [28]. In malignant melanomas, complete regression is observed in 3–15% of primary lesions and 0.08–0.71% of metastatic lesions. Most reports of regression involve cutaneous lesions, and there are no reports of spontaneous regression of PMME. While a combination of immunologic, endocrine, inflammatory and metastatic tumor nutritional factors have been implicated, the underlying mechanisms remain to be elucidated.

Despite its benefits, ICI treatment also has the potential to cause irAEs. The two types of irAEs causing kidney injury - tubulointerstitial nephritis and glomerular disease – have been reported to coexist after nivolumab therapy for other cancers and after ICI treatment for melanoma [29]. Renal pathology in our case also showed signs of both mild tubulointerstitial nephritis and immunoglobulin A (IgA) nephropathy. Full or partial recovery is generally achieved after discontinuation of ICIs and concomitant medications, as in our case [30].

Our patient had metastases to the stomach and bladder, both of which are rare in melanoma patients. Most melanoma lesions arising in the gastrointestinal (GI) tract are metastases. As melanoblasts are only present in the oral cavity, esophagus, and anorectum, the histogenesis of primary melanomas arising in other GI sites remains to be elucidated [31]. Melanoma metastasizing to GI organs is underrecognized, despite autopsies showing melanoma metastases to the esophagus in 4%, stomach in 20%, duodenum in 12%, small bowel in 58%, colon in 22%, rectum in 5%, and liver in 68% of cases [32]. A study of CT scans for metastatic melanoma showed metastases to the esophagus in 7%, stomach in 24%, duodenum in 19%, small bowel in 48%, colon in 4%, liver in 15%, gallbladder in 3%, biliary tree and pancreas in 6%, and mesentery and omentum in 18% of cases [33]. Sensitivity of the CT for small bowel metastases is estimated at 60–70%, and endoscopy may be warranted in suspicious cases. On the other hand, symptoms such as GI bleeding, abdominal pain, and bowel obstruction may only manifest in a small minority of patients with advanced GI metastases [34]. Thus, endoscopic evaluations to rule out metastases should be considered even in the absence of GI symptoms. Stomach and small bowel metastases commonly present as submucosal nodules with central depression presenting a characteristic “bull’s eye” appearance [35]. One case of PMME with gastric metastasis has been reported [36]. A few case reports suggest limited efficacy of immunotherapy for cutaneous melanoma with gastric metastases [37, 38].

Urothelial tumors account for 95% of bladder tumors, while metastases only account for 2.3% [39, 40]. Direct invasion from colorectal, prostate, and cervical cancers are the most common metastatic tumors, with rare distant metastases seen mainly from the stomach, skin, lung, and breast. Metastases almost always present as solitary tumors, and the majority are adenocarcinomas [40]. Both primary and metastatic melanoma of the bladder are extremely rare, accounting for less than 0.1% of bladder tumors and with only about 30 reports of each [40–42]. On the other hand, an autopsy study found bladder metastases in 18% of melanoma patients, suggesting that the latter is more common [32]. Both are generally asymptomatic but may present with painless hematuria [43]. Most metastases come from cutaneous melanoma; there is only one report of PMME metastasizing to the bladder in the English literature [42, 44]. While generally found in the context of widespread metastasis, surgery was performed in 15 cases (including 8 TUR-BT cases) in a review of 24 melanoma cases with metastases to the bladder [42].

One interesting aspect of the bladder metastasis in our case is that it appeared amelanotic despite the primary tumor being melanotic. A Japanese case report also demonstrated an amelanotic bladder metastasis of a melanotic melanoma [45]. In that case as well as ours, histology revealed melanin pigments in the metastatic bladder lesion, and immunohistochemistry was positive for both S-100 and HMB-45. The pathophysiology behind this phenomenon has not been described in the literature and remains to be elucidated.

In conclusion, we report a case of long-term complete remission of metastatic PMME after nivolumab monotherapy. The patient remains tumor-free 55 months after diagnosis and 50 months after the last nivolumab injection. This case sheds light on the prognosis of this rare and dismal disease. More research is warranted to identify factors that increase the likelihood of achieving complete remission with ICIs in PMME as well in as other melanomas.

## Data Availability

Data sharing is not applicable to this article as no datasets were generated or analyzed during the current study.

## Abbreviations

CT: Computed tomography
EGD: Esophagogastroduodenoscopy
GI: Gastrointestinal
ICI: Immune checkpoint inhibitor
IrAE: Immune-related adverse effects
PD-1: Programmed death 1
PMME: Primary malignant melanoma of the esophagus
TUR-BT: Transurethral resection of bladder tumor
